# Theoretical Analysis of Electroconvection in the Electrodialysis Desalination Channel under the Action of Direct Current

**DOI:** 10.3390/membranes12111125

**Published:** 2022-11-10

**Authors:** Aminat Uzdenova, Anna Kovalenko, Makhamet Urtenov

**Affiliations:** 1 Department of Computer Science and Computational Mathematics, Umar Aliev Karachai-Cherkess State University, 369202 Karachaevsk, Russia; 2 Department of Data Analysis and Artificial Intelligence, Kuban State University, 350040 Krasnodar, Russia; 3 Department of Applied Mathematics, Kuban State University, 350040 Krasnodar, Russia

**Keywords:** electrodialysis, desalination, electroconvection, chronopotentiogram, quasi-stationary state, system of Nernst–Planck–Poisson and Navier–Stokes equations

## Abstract

The development of electroconvection in electromembrane systems is a factor that increases the efficiency of the electrolyte solution desalination process. The desalination of the solution, manifested by a change in the distribution of the ion concentration, can affect the mechanisms of development of electroconvection. The purpose of this work is to study the electroconvective flow developing in the desalination channel under various desalination scenarios. The study was carried out on the basis of a mathematical model of the transfer of binary electrolyte ions in the desalination channel formed between the anion and cation exchange membranes under the action of DC current. An analytical estimation of the threshold current density reflecting the conditions of the system transition into a quasi-stationary state has been obtained. The chronopotentiograms of the desalination channel and the thickness of the electroconvective mixing layer are calculated for both pre-threshold and supra-threshold values of the current density.

## 1. Introduction

Electrodialysis is actively used for the production of drinking water in the food and pharmaceutical industries [1,2]. The functioning of electrodialysis systems is determined by the selective properties of ion exchange membranes, which mainly pass ions of one sign (counterions) and prevent the movement of ions of another sign (coions) [3].

One of the methods of electrodialysis system research is chronopotentiometry, that is, studying the change in the potential jump in the system over time under the action of DC current. Based on the analysis of the results of chronopotentiometry, the mechanisms of mass transfer processes and the properties of ion exchange membranes are investigated [4,5,6,7,8]. A typical chronopotentiogram (ChP) of a single ion exchange membrane consists of the following sequence of characteristic sections [4,5,6,7,8]:

1. An initial, almost vertical section, defined by the initial ohmic resistance of the solution;
2. A region of slow growth of the potential jump, which is associated with a decrease in the concentration in the depleted solution near the membrane surface as a result of electrodiffusion processes;
3. At overlimiting current densities, an inflection point of the ChP curve is noted, which is associated with the appearance of another ion transport mechanism [4]; thus, the third transition region of the ChP corresponds to the development of additional ion transport mechanisms;
4. A section where the system is in a stationary or quasi-stationary state (when the potential jump oscillates about a certain fixed value).

Experimental studies [4,5] have shown that for dilute solutions of electrolytes, electroconvection is an additional mechanism responsible for the increase in the intensity of mass transfer and the appearance of an inflection point on the ChP. Electroconvection is understood as the movement of an electrolyte solution under the action of an electric field on a space charge [9,10,11,12]. The difference in the behavior of ions of opposite signs in an ion exchange membrane leads to the formation of a space charge region in a depleted solution near membrane surface, which extends with an increase in the flowing current density [13]. Therefore, at overlimiting current densities, electroconvection significantly affects the transfer of ions in membrane systems with dilute electrolyte solutions [11,14,15,16,17,18,19].

**Figure 1:**
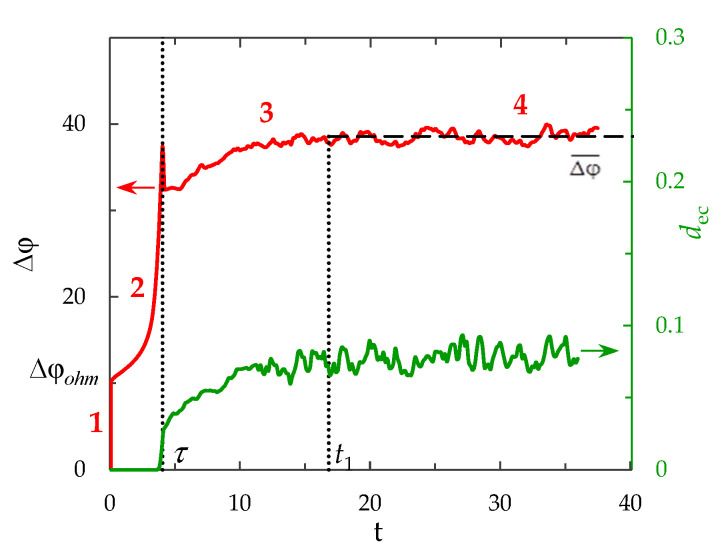
ChP (red line) calculated for the electrolyte solution layer near the surface of the ion exchange membrane, taking into account the development of electroconvection at the overlimiting current density. The dotted lines show the transition time, *τ,* and the approximate time to establish a quasi-stationary state, *t*_1_. The green line represents the dynamics of the averaged over the channel length thickness of the electroconvective mixing layer, *d*_ec_. All quantities are given in dimensionless form (dimensionless voltage unit corresponds to approximately 0.026 V, time unit is 0.066 s, unit of electroconvective mixing layer thickness, *d*_ec_, is 0.25 mm). Adapted from [20].

The theoretical calculation of the ChP for a layer of a dilute electrolyte solution near the surface of a homogeneous ion exchange membrane, taking into account the development of electroconvection, which qualitatively coincides with the experimental data, was performed in [20]. On the calculated ChP, all the characteristic areas described above are distinguished (indicated by numbers in Figure 1). It is shown that the appearance and increase in the electroconvective mixing layer (the plot of this layer thickness in Figure 1 is denoted by *d_ec_*) in the region near the membrane surface corresponds to the transition region of the ChP. Slipping of vortices and vortex complexes along the channel under the action of forced flow causes oscillations in the potential jump. In a quasi-stationary state (in which the potential jump oscillates about certain fixed value), the thickness of the electroconvective mixing layer also becomes quasi-stationary [20]. It was numerically shown that the development of electroconvection significantly reduces the potential jump in the quasi-stationary state of the electrolyte layer near the ion exchange membrane.

Since the ChP was calculated for the electrolyte layer near the surface of a single ion exchange membrane, the outer boundary of this layer is taken as the core of the flow with constant uniform distributions of ion concentrations, flow velocity, and electric potential [20]. This assumption causes the onset of a stationary or quasi-stationary state at any value of the current density, without taking into account the possibility of reducing the concentration in the core of the flow. In this work, numerical simulation of the mass transfer process in the desalination channel formed between the anion exchange (AEM) and cation exchange (CEM) membranes is carried out. The conditions for the establishment of a quasi-stationary state in the desalination channel under the action of DC current are considered, and an analytical estimate of the threshold current density for the transition of the system to the quasi-stationary state is obtained. The ChPs of the desalination channel and the thickness of the electroconvective mixing layer are calculated for both pre-threshold and, for the first time, supra-threshold values of the current density.

## 2. Mathematical Model

**Figure 2:**
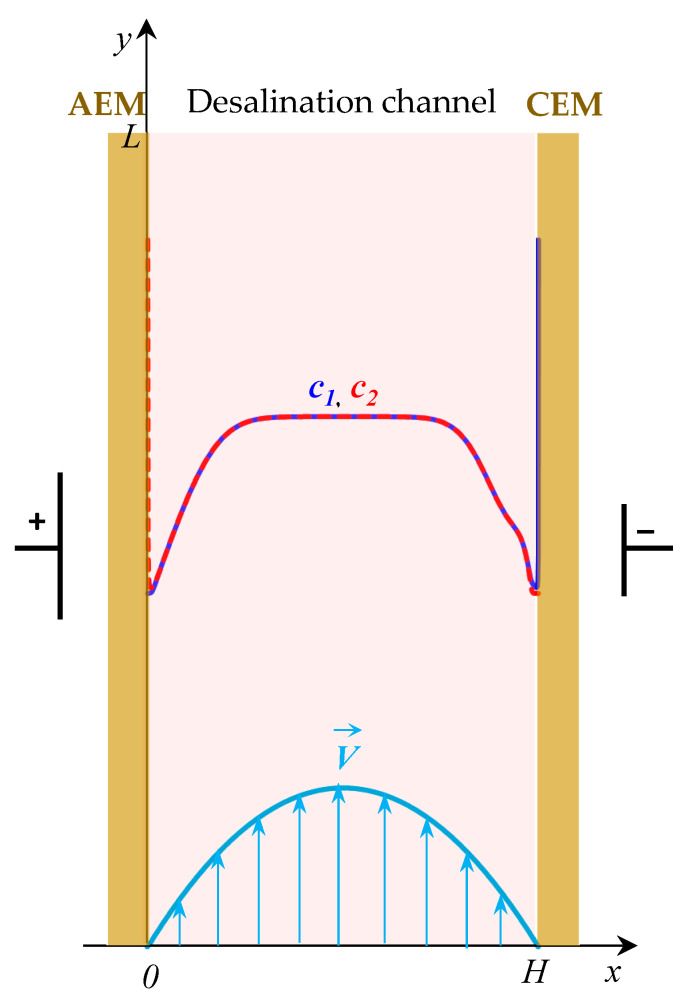
Schematic representation of the simulated system: shown is the desalination channel formed between the anion exchange (AEM) and cation exchange (CEM) membranes; schematically shown is the concentration profiles of cations (*c*_1_, solid line) and anions (*c*_2_, dotted line) in the desalination channel, the velocity of forced electrolyte flow at the inlet to the channel $\vec{V}$.

Consider a binary electrolyte solution in an electrodialysis desalting channel formed between two ion exchange membranes (AEM and CEM), which is pumped at the average velocity *V*_0_ (Figure 2). Let *x* and *y* be the normal and tangent coordinates to the membrane surfaces, respectively; *x* = 0 and *x* = *H* correspond to the interfaces of the electrolyte solution with AEM and CEM; and *y* = 0 and *y* = *L* correspond to the inlet and outlet from the channel (Figure 2).

The non-stationary process of transfer of binary electrolyte ions in membrane systems in the absence of chemical reactions taking into account electroconvection is described by the following equations:

(1) ∂V→∂t+(V→∇)V→=−∇p+1ReΔV→+KelΔφ∇φ, divV→=0,

(2) $${\vec{j}}_{n}^{}=-z_{n}D_{n}^{}c_{n}\nabla \varphi -D_{n}^{}\nabla c_{n}+Pe\;c_{n}\vec{V},\;\;\;n=1,\;2,$$

(3) $$\frac{\partial c_{n}}{\partial t}=-\frac{1}{Pe}\;\mathrm{div}\;{\vec{j}}_{n},\;n=1,\;2,$$

(4) $$-\varepsilon \Delta \varphi =z_{1}c_{1}+z_{2}c_{2}.$$

Equations (1)–(4) are given in a dimensionless form: time, *t*, is scaled by the value $H/V_{0}$; spatial coordinates, *x* and *y*, by the channel width *H*; velocity, $\vec{V}$, by the average velocity of the forced flow *V*_0_; pressure, *p*, by the velocity head $\rho V_{0}^{2}$; the concentration of the *n*-th ion, $c_{n}$, by the input concentration of the electrolyte *c*_0_; electric potential, φ, by the value RT/F; the diffusion coefficient of the *n*-th ion, *D_n_*, by the electrolyte diffusion coefficient $D=D_{1}D_{2}\left(z_{1}-z_{2}\right)/\left(D_{1}z_{1}-D_{2}z_{2}\right)$; and the flux of ions of the *n*-th kind, ${\vec{j}}_{n}$, by the value $Dc_{0}/H$. Here, Re=V0H/ν is the Reynolds number, $Pe=V_{0}H/D$ is the Peclet number, $\varepsilon =RT\varepsilon_{0}\varepsilon_{r}/\left(c_{0}F^{2}H^{2}\right)$ and $K_{el}=\varepsilon_{0}\varepsilon_{r}R^{2}T^{2}/\left(\rho_{0}V_{0}^{2}F^{2}H^{2}\right)$ are the dimensionless parameters [21]; *Z_n_* is the charge number of the *n*-th ion; *F* is the Faraday constant; *R* is the gas constant; *T* is the absolute temperature; $\varepsilon_{0}$ is the electrical constant; $\varepsilon_{r}$ is the relative permittivity of the solution; $\rho_{0}$ is the density of the solution; ν is the kinematic viscosity; and $\varepsilon_{r}$, $\rho_{0}$ and ν are assumed to be constant.

The quantities $\vec{V},\;p,\;{\vec{j}}_{1},\;{\vec{j}}_{2},\;c_{1},\;c_{2},\;\varphi$ are unknown functions of time *t* and spatial coordinates *x* and *y*. The Navier–Stokes equations, Equation (1), describe the velocity field under the action of a forced flow and a force of the electric field. The equations of Nernst-Planck, Equation (2), material balance, Equation (3), and Poisson, Equation (4) describe the concentration of ions and the electric potential.

Equation (5) defines the total current density, $\vec{i}$, including the Faraday current (conduction current), ${\vec{i}}_{F}=z_{1}{\vec{j}}_{1}^{}+z_{2}{\vec{j}}_{2}^{}$, and charging current (displacement current), ${\vec{i}}_{c}=-\varepsilon \;Pe\;\frac{\partial }{\partial t}(\nabla \varphi )$, [22]:

(5) $$\vec{i}=z_{1}{\vec{j}}_{1}^{}+z_{2}{\vec{j}}_{2}^{}-\varepsilon \;Pe\;\frac{\partial }{\partial t}(\nabla \varphi ).$$

When modeling the DC current mode, the conditions of constancy of the average current density at the solution/membrane interfaces, *i_av_*, must be satisfied [23]:

(6) $$i_{av}=\frac{1}{L}\int_{0}^{L}i_{x}(0,y,t)dy=\frac{1}{L}\int_{0}^{L}i_{x}(1,y,t)dy.$$

The vector field of the total current density is solenoidal, $div\;\vec{i}=0$, so the electric current function, η, can be defined as follows [23]:

(7) $$i_{x}=\frac{\partial \eta }{\partial y},\;i_{y}=-\frac{\partial \eta }{\partial x}.$$

On the basis of Equations (2), (5) and (7), Equation (8) was obtained, which makes it possible to determine the current density distribution in the DC current mode [20]:

(8) $$\begin{array}{l} \Delta \eta =-\left(\left(z_{1}^{2}D_{1}\frac{\partial c_{1}}{\partial y}+z_{2}^{2}D_{2}\frac{\partial c_{2}}{\partial y}\right)\frac{\partial \varphi }{\partial x}-\left(z_{1}^{2}D_{1}\frac{\partial c_{1}}{\partial x}+z_{2}^{2}D_{2}\frac{\partial c_{2}}{\partial x}\right)\frac{\partial \varphi }{\partial y}\right)+\\ +Pe\left(z_{1}\frac{\partial c_{1}}{\partial y}+z_{2}\frac{\partial c_{2}}{\partial y}\right)V_{x}-Pe\left(z_{1}\frac{\partial c_{1}}{\partial x}+z_{2}\frac{\partial c_{2}}{\partial x}\right)V_{y}+Pe\left(z_{1}c_{1}+z_{2}c_{2}\right)\left(\frac{\partial V_{x}}{\partial y}-\frac{\partial V_{y}}{\partial x}\right) \end{array}$$

The numerical solution of the boundary value problem of the model requires the definition of the boundary conditions for Equations (1), (3), (4) and (8). Let us set boundary conditions similar to the conditions in [20] with the difference that the entire desalination channel from AEM to CEM is considered.

At the channel inlet, x∈[0, 1], *y* = 0, the parabolic Poiseuille velocity profile is taken, Equation (9); for ion concentrations, the Dankwerts conditions are taken, which determine the fact that the rate of entry of ions into the channel is equal to the rate at which ions cross the plane *y* = 0 through a combination of electromigration, diffusion, and convection, Equation (10); and the condition for the electric potential obtained from Equations (2) and (6) taking into account the zero density of the tangential current, *i_y_*(*x*, 0, t) = 0 (the tangential component of the displacement current is negligibly small), Equation (11):

(9) $$V_{x}\left(x,0,t\right)=0,\;V_{y}\left(x,0,t\right)=6x(1-x),$$

(10) $$\left(-z_{n}D_{n}c_{n}\frac{\partial \varphi }{\partial y}-D_{n}\frac{\partial c_{n}}{\partial y}+Pec_{n}V_{y}\right)\left(x,0,t\right)=Pec\prime V_{y}(x,0,t),\;n=1,\;2,$$

(11) $$\frac{\partial \varphi }{\partial y}\left(x,0,t\right)=-\frac{1}{z_{1}^{2}D_{1}+z_{2}^{2}D_{2}}\left(z_{1}D_{1}\frac{\partial c_{1}}{\partial y}+z_{2}D_{2}\frac{\partial c_{2}}{\partial y}\right).$$

At the channel outlet, x∈[0, 1], *y* = *L*, the "soft" boundary conditions are accepted [24], Equation (12); tangential derivatives of ion concentrations and potential are set equal to zero, Equations (13) and (14):

(12) $$V_{x}\left(x,L,t\right)=0,\;\frac{\partial V_{y}}{\partial y}\left(x,L,t\right)=0,$$

(13) $$\frac{\partial c_{n}}{\partial y}\left(x,L,t\right)=0,\;n=1,\;2,$$

(14) $$\frac{\partial \varphi }{\partial y}\left(x,L,t\right)=0.$$

At the solution/CEM interface, *x* = 1, y∈[0, L], the no-slip condition is applied, Equation (15); the counterion concentration, *c*_1_, is set as a constant value that is *N_c_* times greater than the input concentration of solution [13], Equation (16); continuous flux of coions, where the transfer numbers of ions in membranes, *T_n_*_A_ and *T_n_*_C_, determine the portion of the current carried by ions of this type (moreover, *T*_1A_ + *T*_2A_ = 1 and *T*_1C_ + *T*_2C_ = 1) [25], Equation (17); the normal derivative of the electric field potential is given as a function of the electric current density ($i_{x}=\partial \eta /\partial y$), Equation (18) [26]:

(15) $$V_{x}\left(1,y,t\right)=0,\;V_{y}\left(1,y,t\right)=0,$$

(16) $$c_{1}\left(1,y,t\right)=N_{c},$$

(17) $$\left(-D_{2}\frac{\partial c_{2}}{\partial x}-z_{2}D_{2}c_{2}\frac{\partial \varphi }{\partial x}\right)\left(1,y,t\right)=\frac{T_{2C}}{z_{2}}i_{x}\left(1,y,t\right),$$

(18) $$\frac{\partial \varphi }{\partial x}\left(1,y,t\right)=-\left(\frac{\left(\frac{\partial \eta }{\partial y}+z_{1}D_{1}\frac{\partial c_{1}}{\partial x}+z_{2}D_{2}\frac{\partial c_{2}}{\partial x}\right)}{z_{1}^{2}D_{1}c_{1}+z_{2}^{2}D_{2}c_{2}}\right)(1,y,t).$$

The similar conditions are accepted for the velocity and concentrations of ions at the solution/AEM interface, *x* = 0, y∈[0, L]:

(19) $$V_{x}\left(0,y,t\right)=0,\;V_{y}\left(0,y,t\right)=0,$$

(20) $$c_{2}\left(0,y,t\right)=N_{a},$$

(21) $$\left(-D_{1}\frac{\partial c_{1}}{\partial x}-z_{1}D_{1}c_{1}\frac{\partial \varphi }{\partial x}\right)\left(0,y,t\right)=\frac{T_{1A}}{z_{1}}i_{x}\left(0,y,t\right).$$

In the system of Equations (1)–(4), (8) the electric potential enters only in the form of derivatives on spatial coordinates; therefore, only the potential jump is significant. For the convenience of calculations, we set the zero potential on the left boundary, Equation (22):

(22) φ(0,y,t)=0.

Boundary conditions for the electrical current function [23]:

(23) $$\frac{\partial \eta }{\partial x}\left(0,y,t\right)=0,\;\frac{\partial \eta }{\partial x}\left(1,y,t\right)=0,\;\eta \left(x,0,t\right)=0,\;\eta \left(x,l,t\right)=i_{av}l.$$

At the initial time, *t* = 0, the flow is parabolic, Equation (24); the electrical neutrality condition is satisfied at all points of the desalination channel, and the ion concentrations are equal to the initial electrolyte concentration *c*_0_, Equation (25); the potential is zero everywhere, Equation (26):

(24) $$V_{x}\left(x,y,0\right)=0,\;V_{y}\left(x,y,0\right)=6x(1-x),$$

(25) $$c_{1}\left(x,y,0\right)=1,\;c_{2}\left(x,y,0\right)=1,$$

(26) φ(x,y,0)=0.

## 3. Results and Discussion

### 3.1. The Condition for the Transition of the System to a Quasi-Stationary State

To determine the condition for the transition of the electrolyte solution in the desalination channel to a quasi-stationary state, we perform a sequence of transformations of the equation for the cation concentration, Equation (3):

(27) $$\frac{\partial c_{1}(x,y,t)}{\partial t}=-\frac{1}{Pe}\;\left(\frac{\partial j_{1x}(x,y,t)}{\partial x}+\frac{\partial j_{1y}(x,y,t)}{\partial y}\right).$$

We multiply Equation (27) by the charge number of the cation, *z*_1_, and perform the integration over the considered region of the desalination channel:

(28) $$\frac{\partial }{\partial t}\int_{0}^{1}\int_{0}^{L}z_{1}c_{1}(x,y,t)dxdy=-\frac{1}{Pe}\;\int_{0}^{1}\int_{0}^{L}z_{1}\frac{\partial j_{1x}(x,y,t)}{\partial x}dxdy-\frac{1}{Pe}\;\int_{0}^{1}\int_{0}^{L}z_{1}\frac{\partial j_{1y}(x,y,t)}{\partial y}dxdy.$$

The first term on the right side of Equation (28) is determined taking into account the boundary conditions (17) and (21), and the fact that $T_{1C}+T_{2C}=1$:

(29) $$\begin{array}{l} -\frac{1}{Pe}\;\int_{0}^{1}\int_{0}^{L}z_{1}\frac{\partial j_{1x}(x,y,t)}{\partial x}dxdy=-\frac{1}{Pe}\;\int_{0}^{L}\left[z_{1}j_{1x}(1,y,t)-z_{1}j_{1x}(0,y,t)\right]dy=\\ =-\frac{1}{Pe}\;\int_{0}^{L}\left[\left(1-T_{2C}\right)i_{x}(1,y,t)-T_{1A}i_{x}(0,y,t)\right]dy=-\frac{L}{Pe}\left(1-T_{2C}-T_{1A}\right)i_{av} \end{array}$$

In the last transformation (29), the conditions for the constancy of the average current density at the solution/membrane interfaces, Equation (6), are taken into account.

The second term on the right side of Equation (27) is determined taking into account the boundary conditions (9)–(14):

(30) $$\begin{array}{l} -\frac{z_{1}}{Pe}\;\int_{0}^{1}\int_{0}^{L}\frac{\partial j_{1y}(x,y,t)}{\partial y}dxdy=-\frac{z_{1}}{Pe}\;\int_{0}^{1}\left[j_{1y}(x,L,t)-j_{1y}(x,0,t)\right]dx=\\ =-\frac{z_{1}}{Pe}\int_{0}^{1}\left[Pec_{1}(x,L,t)V_{y}(x,L,t)-Pe6x(1-x)\right]dx=-z_{1}\int_{0}^{1}c_{1}(x,L,t)V_{y}(x,L,t)dx+z_{1} \end{array}$$

Then, Equation (27) can be written as:

(31) $$\frac{\partial }{\partial t}\int_{0}^{1}\int_{0}^{L}z_{1}c_{1}(x,y,t)dxdy=-\frac{L}{Pe}\left(1-T_{2C}-T_{1A}\right)i_{av}-z_{1}\int_{0}^{1}c_{1}(x,L,t)V_{y}(x,L,t)dx+z_{1}.$$

The terms on the right side of Equation (31) are the values of the partial current of cations through the boundaries of the system under consideration. The condition for the transition of the system to a stationary (or quasi-stationary) state is the equality to zero (or smallness) of the right side of Equation (31):

(32) $$-\frac{L}{Pe}\left(1-T_{2C}-T_{1A}\right)\bar{i_{av}}-z_{1}\;\int_{0}^{1}\bar{\;c_{1}}(x,L)V_{y}(x,L)dx+z_{1}=0,$$

where $\bar{i_{av}}$ is the current density and $\bar{\;c_{1}}(x,L)\;$ is the concentration distribution at the outlet of the channel in the stationary state (or their time-averaged values in the quasi-stationary state). The cation flux at the channel outlet in the quasi-stationary state decreases with increasing current density:

(33) $$z_{1}\;\int_{0}^{1}\bar{\;c_{1}}(x,L)V_{y}(x,L)dx=z_{1}-\frac{L}{Pe}\left(1-T_{2C}-T_{1A}\right)\bar{i_{av}}.$$

The zero flux of cations at the outlet of the channel determines the balance state of the processes of desalination of the electrolyte solution and its entry into the channel through forced flow. The current density for these conditions is determined by Equation (34):

(34) $$i_{tr}=\frac{z_{1}Pe}{L\left(1-T_{2C}-T_{1A}\right)},$$

or in dimensional form (here $L^{d}$ is the dimensional length of the channel):

(35) $$i_{tr}^{d}=\frac{z_{1}Fc_{0}V_{0}H}{L^{d}\left(1-T_{2C}-T_{1A}\right)}.$$

Therefore, Equation (34) defines the threshold current density for the transition of the system to the quasi-stationary state, *i_tr_*: at value of current density less than *i_tr_* the system goes into the stationary (or quasi-stationary at overlimiting currents) state, and when the value of current density is exceeded *i_tr_*, the electrolyte is completely depleted, and the potential jump increases rapidly.

Note that Equation (32) makes it possible to estimate the current efficiency (*CE*) and the energy per ion removal (*EPIR*) in the DC current mode. *CE* shows how efficiently the current is used to remove ions [27]:

(36) $$CE=\frac{Pe\;\int_{0}^{1}\left[z_{1}j_{1y}(x,0,t)-z_{1}j_{1y}(x,L,t)\right]dx}{i_{av}L}=\frac{Pe\;z_{1}\;}{i_{av}L}\left(1-\;\int_{0}^{1}\;c_{1}(x,L,t)V_{y}(x,L,t)dx\right).$$

*EPIR* is determined by Equation (37) (scaled by *RT*) [27]:

(37) $$EPIR=\frac{i_{av}\;L\;\Delta \varphi }{Pe\;\int_{0}^{1}\left[z_{1}j_{1y}(x,0,t)-z_{1}j_{1y}(x,L,t)\right]dx}=\frac{i_{av}\;L\;\Delta \varphi }{Pez_{1}\;\left(1-\;\int_{0}^{1}\;c_{1}(x,L,t)V_{y}(x,L,t)dx\right)}.$$

For a quasi-stationary state, taking into account Equation (32), it can be written:

(38) $$CE=1-T_{2C}-T_{1A},$$

(39) $$EPIR=\frac{\bar{\Delta \varphi }}{1-T_{2C}-T_{1A}}.$$

where $\bar{\Delta \varphi }$ is the potential jump in the stationary state or the time-averaged value in the quasi-stationary state. *EPIR* in dimensional form (J/mol):

(40) $$EPIR^{d}=\frac{F\bar{\Delta \varphi^{d}}}{1-T_{2C}-T_{1A}}.$$

Thus, in the quasi-stationary state of desalination by DC current *CE* < 1 due to imperfect membrane selectivity, *EPIR* is determined by the potential jump and ion transport numbers in membranes.

### 3.2. Numerical Simulation

The numerical solution of problem (1)–(26) is found by the finite element method [28]. The calculations are performed for the NaCl solution at dimensionless numbers Re = 0.112, *Pe* = 62, $\varepsilon =7.6\cdot 10^{-8}$, $K_{el}=0.047$, $T_{1A}=T_{2C}=0.03$, which corresponds to the following values of the system parameters: $c_{0}=10^{-2}$ mol/m^3^, $H=0.5\cdot 10^{-3}$ m, $V_{0}=2\cdot 10^{-4}$ m/s, $D_{1}=1.33\cdot 10^{-9}$ m^2^/s, $D_{2}=2.05\cdot 10^{-9}$ m^2^/s. For the forced flow velocity, $V_{0}$, a small value was chosen so that calculations for the supra-threshold regime could be performed at low current densities (which are characterized by less computational complexity).

When analyzing the results of studying processes in membrane systems, it is customary to normalize the current density by the value of the diffusion limiting current density, *i_lim_*. For the solution under consideration (NaCl) we use the limiting current density of the CEM defined by Equation (41) [29]:

(41) $$i_{\lim}=\frac{1}{T_{1C}-t_{1}}\left[1.47{\left(\frac{H^{2}V_{0}}{LD}\right)}^{1/3}-0.2\right],$$

where *t*_1_ is the transport number of the cation in solution, *t*_1_ = 0.395 [30].

The limiting current density, *i_lim_*, determines the state of almost complete depletion of the ion concentration at the solution/CEM interface. When the limiting current density *i_lim_* is exceeded, an extended space charge region is formed and an electroconvective flow develops in the region near the CEM surface [20,28]. When the current density is higher than $i_{\lim}\left(T_{1C}-t_{1}\right)/\left(T_{2A}-t_{2}\right)\approx 1.58i_{\lim}$ (where *t*_2_ = 0.605 is the anion transport number in solution), similar processes develop in the region near the AEM surface.

**Figure 3:**
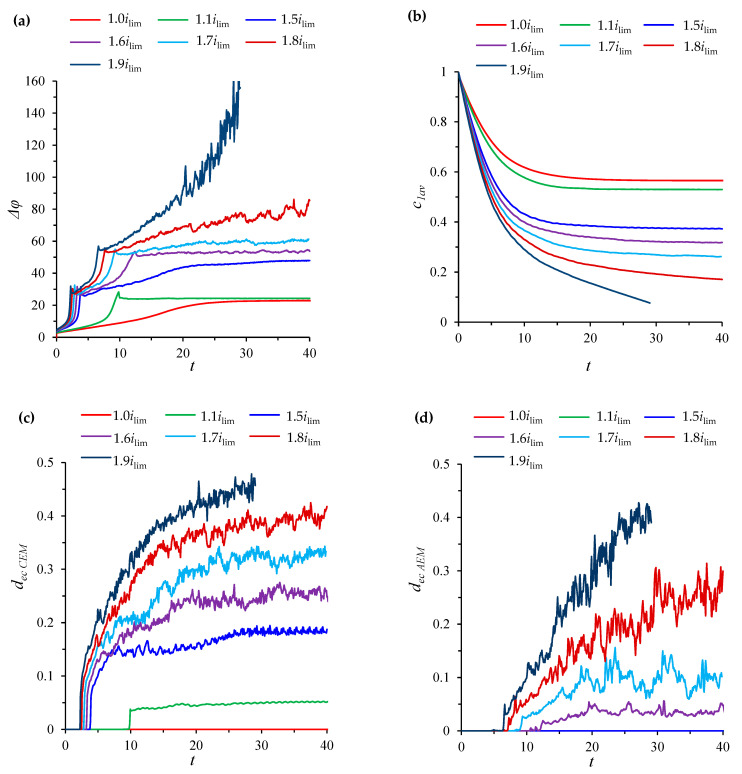
ChPs (**a**), time dependencies of the average concentration of cations in the desalination channel, $c_{1av}(t)=1/L\int_{0}^{1}\int_{0}^{L}c_{1}(x,y,t)dxdy$, (**b**), average thicknesses of the electroconvective mixing layer *d*_ec CEM_ (**c**) and *d*_ec AEM_ (**d**). Calculation results for current density *i_av_/i_lim_*= 1, 1.1, 1.5, 1.6, 1.7, 1.8, 1.9. All quantities are given in dimensionless form (dimensionless voltage unit corresponds to approximately 0.026 V, time unit is 2.5 s, ion concentration unit is 0.01 mol/m^3^, unit of electroconvective mixing layer thickness is 0.5 mm).

Experimental studies of electrodialysis systems, as a rule, are carried out under conditions when the threshold current density of the quasi-stationary state, *i_tr_*, is greater than the values of the limiting current density for CEM and AEM, that is $i_{\lim}<i_{\lim}\left(T_{1C}-t_{1}\right)/\left(T_{2A}-t_{2}\right)<i_{tr}$. For example, the system settings described above at channel length *L* = 8 correspond to these conditions. Then, the threshold current density calculated by Equation (34) will be equal to *i_tr_* ≈ 1.76*i_lim_*. Figure 3 shows ChPs calculated for the following values of the current density *i_av_/i_lim_*: 1, 1.1, 1.5, 1.6, 1.7, 1.8, 1.9.

**Figure 4:**
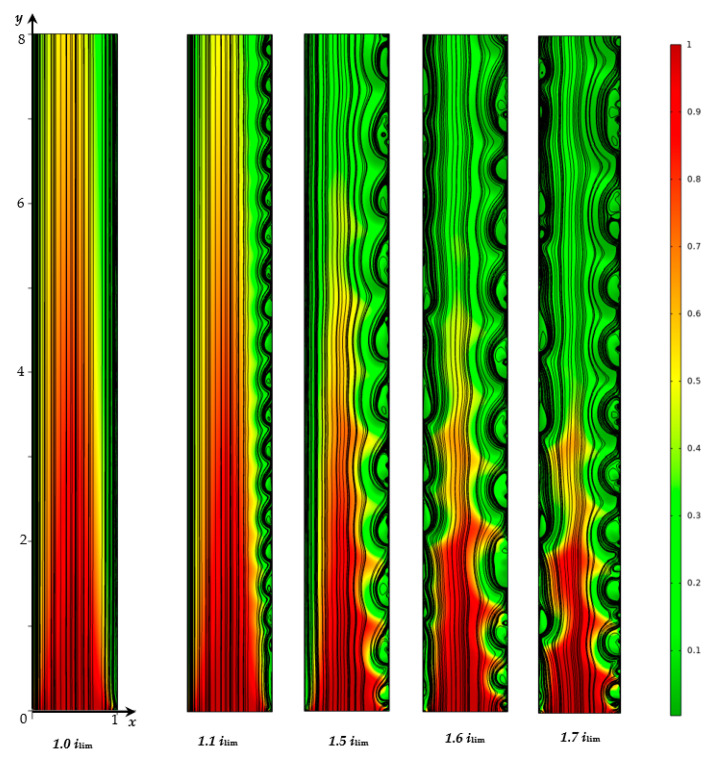
Solution streamlines (black lines) and cation concentration distribution (shown in color) in the quasi-stationary state (*t* = 40, which corresponds to 100 s), calculated for current density values $i_{av}/i_{\lim}$ = 1, 1.1, 1.5, 1.6, 1.7.

At the limiting current density, *i_av_* = *i_lim_*, at the ChP of the system under consideration, there is a successive change in the initial ohmic region by a region of slow growth of the potential jump (associated with a decrease in concentration in the regions near the surfaces of both membranes) and a transition to a stationary state without the development of electroconvection (Figure 4).

At values of the current density above the limiting value, *i_lim_*, electroconvection develops in the region near the CEM, and an electroconvective mixing layer is formed (see Figure 4). In order to quantitatively characterize the intensity of electroconvection, the averaged over the channel length thickness of the electroconvective mixing layer, *d*_ec_, is calculated. The boundary of the electroconvective mixing layer was determined as a point at which the difference in the root-mean-square value of the velocity in calculations with and without taking into account electroconvection exceeds 15% of the average forced flow velocity, *V*_0_, (by analogy with [31]). In the considered case of a desalination channel with two membranes, the thickness of the electroconvective mixing layer is calculated for the channel halves at the CEM (*d*_ec CEM_) and at the AEM (*d*_ec AEM_), Figure 3c,d.

On the ChPs for the current density equal to 1.1*i_lim_* and 1.5*i_lim_*, at the moment of development of the electroconvective mixing layer near the surface of CEM, a decrease in the growth rate of the potential jump and a transition to a quasi-stationary state, accompanied by an increase in *d*_ec CEM_, are observed. The shapes of these curves differ significantly in the transition region, since as the current density approaches the value of the limiting state of the solution at AEM, $i_{\lim}\left(T_{1C}-t_{1}\right)/\left(T_{2A}-t_{2}\right)$, the effect of desalination in this region increases. The ChPs for the current density, which exceeds the limiting value for the CEM, but are less than the limiting current value for the AEM, differs from the ChPs for the underlimiting and limiting densities by the appearance of a transition section, which corresponds to an increase in the thickness of the electroconvective mixing layer before the transition to the quasi-stationary state. Thus, the ChPs for the current density in the range $i_{\lim}<i_{av}<i_{\lim}\left(T_{1C}-t_{1}\right)/\left(T_{2A}-t_{2}\right)$ contain all the regions characteristic of a single membrane, observed experimentally [4,5,6,7,8].

On the curve of the thickness *d*_ec CEM_ for the current density of 1.1*i_lim_*, there are periodic oscillations of small amplitude (≈0.001 in the quasi-stationary state), which are associated with the movement of single electroconvective vortices (Figure 4) along the membrane surface from the inlet to the outlet of the desalination channel under the action of forced electrolyte flow. Thickness *d*_ec CEM_ fluctuations for the current density of 1.5*i_lim_* have a more complex shape and a larger amplitude (≈0.012 in the quasi-stationary state), which is due to the fact that instead of single vortices, larger vortex complexes are observed (Figure 4).

At current densities of 1.6*i_lim_*, 1.7*i_lim_*, 1.8*i_lim_*, and 1.9*i_lim_* (which exceed the value of the limiting state density at AEM, $i_{\lim}\left(T_{1C}-t_{1}\right)/\left(T_{2A}-t_{2}\right)$), an electroconvective flow also develops in the region near the surface of the AEM (Figure 3d and Figure 4), while at the ChPs, there is a second slowdown in the growth of the potential jump. Thus, ChPs for the current density, which exceeds the limiting value for the AEM, $i_{\lim}\left(T_{1C}-t_{1}\right)/\left(T_{2A}-t_{2}\right)$, but is less than the threshold value, *i_tr_*, differ from the one described above in that the transition state in which the electroconvective flow develops is divided into two fragments, one of which is associated with the appearance of electroconvective vortices in the region near the CEM, and the second one, in the region near the surface of the AEM. With an increase in current density, an increase in the size of electroconvective vortex complexes is fixed, accompanied by an increase in the amplitude of electroconvective mixing layers’ thickness fluctuations at the CEM (*d*_ec CEM_) and at the AEM (*d*_ec AEM_).

At a current density equal to 1.8*i_lim_* and 1.9*i_lim_* (which exceed the threshold value, *i_tr_*), all the characteristic sections of the ChPs described above are observed, except for the quasi-stationary state. Desalination of the solution occurs until the concentration is completely depleted (Figure 3b), while an increase in the potential jump is observed.

**Figure 5:**
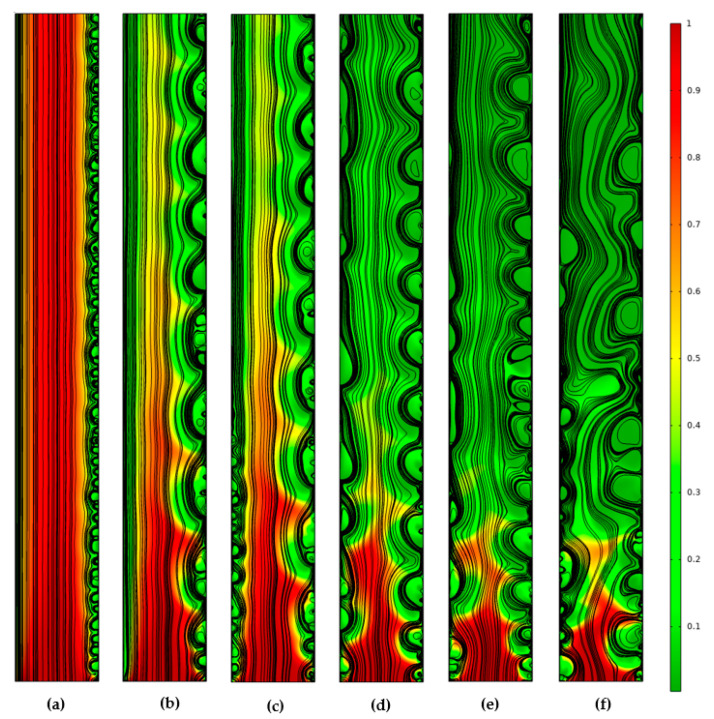
Solution streamlines (black lines) and cation concentration distribution (shown in color) at *t* = 2.4 (**a**), 6.4 (**b**), 6.8 (**c**), 14 (**d**), 20 (**e**), 24.8 (**f**) (dimensionless unit of time corresponds to 2.5 s), calculated for current density *i_av_*/*i_lim_* = 1.9.

Changes in the structure of the electroconvective flow in the desalination channel for the current density 1.9*i_lim_* can be seen in Figure 5. The appearance of electroconvection near the CEM occurs almost simultaneously along the entire membrane surface, which is associated with the relative homogeneity (in the longitudinal direction) of solution depletion in this region (Figure 5a). The appearance of electroconvection near the AEM surface occurs later at a higher degree of solution desalination (Figure 3b). The ion concentration field at this point in time (Figure 5c) is characterized by large concentration field gradients in the region at the channel inlet compared to the region at the channel outlet. Therefore, for the considered parameters of the system, electroconvection near AEM is developed earlier in the region near the channel inlet. This explains the rapid growth of *d*_ec CEM_ and the more gentle growth of *d*_ec AEM_ immediately after the development of electroconvection. The depletion of the electrolyte solution in the region near the channel outlet is accompanied by the destruction of the space charge regions and, consequently, by a decrease in electroconvection (Figure 5f) [32,33].

On all ChPs, for a current density above the limiting value, potential jump oscillations are observed, which appear almost simultaneously with the development of electroconvection (Figure 3a,c,d). This is due to the non-stationary dynamics of vortices (slip of vortices in the tangential direction or changes in the structure of the vortex complex). The non-stationary dynamics of vortices cause fluctuations in the ion concentration, and, consequently, the space charge density in the region of the electroconvective mixing layer, which determines the fluctuations in the potential jump. Similar fluctuations in the potential jump and the size of electroconvective vortices were recorded in a direct experimental study of electroconvection near the surface of an ion exchange membrane under the action of DC current in [34].

**Figure 6:**
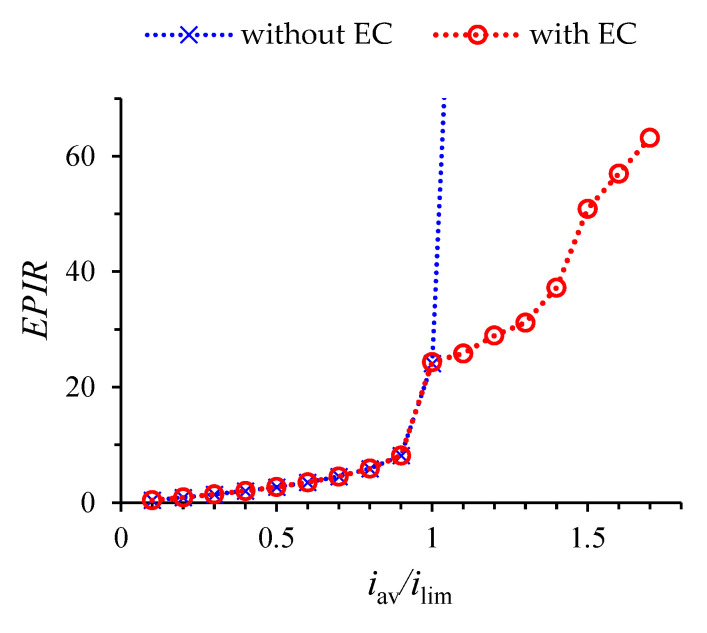
Energy per ion removal in a quasi-stationary state, calculated with and without taking into account the development of electroconvection (EC) for the following values of current density *i_av_*/*i_lim_*: 0, 0.1, …, 1.7.

Figure 6 shows the results of calculations of the *EPIR* (by Equation (39)), performed with and without taking into account the development of electroconvection for the following values of current density *i_av_*/*i_lim_*: 0, 0.1, …, 1.7. Figure 6 shows that *EPIR* increases with increasing current density. Exceeding the limiting density in the calculation without taking into account electroconvection is accompanied by a sharp increase in *EPIR*. The appearance of electroconvection first at the CEM (at *i_lim_*) and later at the AEM (see the value at 1.6*i_lim_*) significantly reduces *EPIR*. Therefore, the development of electroconvection significantly reduces the *EPIR*. At the supra-threshold current density, the sharp increase in the potential jump causes large *EPIR*.

## 4. Conclusions

The study of the desalination process of the electrolyte solution in the electrodialysis desalination channel under the action of DC current by means of numerical simulation is carried out. The model makes it possible to describe the development of electroconvection caused by the action of an electric field on the space charge formed near the surface of ion exchange membranes, taking into account the process of desalting the electrolyte solution. The analytical estimate of the threshold current density of the transition to a quasi-stationary state is derived. ChPs calculated for pre-threshold current densities are characterized by a transition to a quasi-stationary state over time. The thickness of the electroconvective mixing layer under these conditions is also a quasi-stationary quantity. The ChP for supra-threshold current densities reflects an unlimited growth of the potential jump with a gradual depletion of the ion concentration. In supra-threshold current modes, electroconvection covers the entire channel over time. The depletion of the electrolyte concentration causes the destruction of the space charge regions and the attenuation of electroconvection. It is shown that in pre-threshold current regimes, the development of electroconvection significantly reduces the energy spent on ions’ removing.

## Data Availability

Not applicable.
